# Geographic Distribution and Temporal Trends of HIV-1 Subtypes through Heterosexual Transmission in China: A Systematic Review and Meta-Analysis

**DOI:** 10.3390/ijerph14070830

**Published:** 2017-07-24

**Authors:** Peipei Xiao, Jianjun Li, Gengfeng Fu, Ying Zhou, Xiping Huan, Haitao Yang

**Affiliations:** 1 Department of Epidemiology and Health Statistics, School of Public Health, Southeast University, Nanjing 210009, China; xiaopei_seu@126.com; 2 Department of HIV/STD Prevention and Control, Jiangsu Provincial Center for Disease Prevention and Control, Nanjing 210009, China; babbittlee@gmail.com (J.L.); fugf@jscdc.cn (G.F.); vera_46@sina.com (Y.Z.); huanxp@vip.sina.com (X.H.); 3 Jiangsu Research Institute of Schistosomiasis Control, Wuxi 214064, China

**Keywords:** HIV-1, subtypes, heterosexual transmission, China

## Abstract

*Background*: Heterosexual transmission (HST) has become the current predominant transmission pathways of the HIV-1 epidemic in China. The aim of this study was to explore the geographic and dynamic change of HIV-1 subtypes through HST in China from published studies. *Methods*: Several electronic databases were searched to identify the studies, and the overall prevalence of HIV-1 subtypes was estimated by a meta-analysis method. Subgroup analysis was conducted by study region and time period. Publication bias was evaluated using Egger’s test. The *χ*^2^ test was used to evaluate the proportion differences among subgroups. Sensitivity analysis was carried out to assess the stability of the overall prevalence estimates. *Results:* 42 studies were included in our final analysis. The overall prevalence of CRF01_AE was 46.34% (95% CI: 40.56–52.17%), CRF07_BC was 19.16% (95% CI: 15.02–23.66%), B/B’ was 13.25% (95% CI: 9.68–17.25%), CRF08_BC was 10.61% (95% CI: 7.08–14.70%), and C was 4.29% (95% CI: 1.85–7.48%). In subgroup analysis, the prevalence of CRF01_AE and CRF07_BC increased, while the prevalence of B/B’ decreased over time, whereby the prevalence of CRF07_BC and CRF08_BC have exceeded that of B/B’ since 2010. A significant higher prevalence of CRF01_AE was found in the South provinces, CRF07_BC in East provinces, CRF08_BC and C in Southwest provinces, and B/B’ in North provinces. *Conclusions*: The HIV-1 prevalent strains have evolved into complicated and diverse subtypes, and the proportion of HIV-1 subtypes through HST has changed constantly in different regions and periods in China. This highlights the urgent need to vigorously strengthen the prevention and control of the HIV-1 epidemic.

## 1. Introduction

The history of HIV infection in China was first documented in a foreign tourist with AIDS and four Chinese hemophiliac patients in 1985 [1]. Originally, the HIV epidemic was largely confined to certain high-risk populations, such as intravenous drug users (IDUs) and former plasma donors (FPDs) in geographically disparate areas [2,3]. However, in the following several decades, HIV has gradually spread to the general population, and the main drivers of HIV epidemic in China have shifted considerably from blood transmission to sexual contact transmission [4]. By the end of 2016, it was reported that 664,751 people were infected with HIV/AIDS and that 124,555 new infections had occurred. Newly identified HIV/AIDS cases caused by sexual transmission have accounted for 94.7% of all the newly identified cases, and among those infected by sexual transmission, the proportion of heterosexual transmission (HST) has dramatically increased from 8.7% in 2008 to 67.1% in 2016 [5,6]. Therefore, HST has become the predominant factor of the current HIV epidemic in China.

High mutation and recombination contribute to an extensive diversity of HIV variants [7]. The two distinct types of virus are HIV-1 and HIV-2, HIV-1 is dispersed worldwide, whereas HIV-2 is mainly restricted to West Africa [8]. HIV-1 genotypes are classified into four genetic groups: M, N, O and P [9], among which, the vast majority (more than 90%) of HIV infections attribute to HIV-1 group M. Group M is further divided into nine genetic subtypes (A–D, F–H, J, and K), six sub-subtypes (A1–A4, and F1–F2) and a variety of recombinant forms [10,11]. Unique recombinant forms (URFs) that succeed in being circulating in three or more epidemiologically unlinked individuals are called circulating recombinant forms (CRFs) [12]. To date, 81 CRFs have been reported from Los Alamos National Laboratory HIV Sequence Database (http://www.hiv.lanl.gov/content/sequence/HIV/ CRFs/CRFs.html). Apart from those CRFs, innumerable URFs have also been described. More than that, a number of CRFs have recombined further with other subtypes or CRFs leading to so-called second generation recombinants (SGRs) [13].

In China, the distribution of HIV-1 subtypes is highly diverse and complex. The latest nationwide molecular epidemiological survey indicated that over 11 genetic variants are circulating among people infected with HIV-1. CRF01_AE, CRF07_BC, CRF08_BC, and subtype B account for 92.8% of HIV-1 variants [14]. Furthermore, the survey also showed different HIV-1 subtypes were associated with different geographical regions and modes of transmission. All the above-mentioned four HIV-1 strains occurred in significant proportions among the HST population, suggesting an extension of the HIV-1 epidemic from high-risk populations into the general population. The high complexity of the HIV-1 epidemic underscores the serious challenges in designing effective prevention measures against HIV transmission. Additionally, HIV-1 subtypes tend to be associated with fast HIV progression, drug resistance, and virologic responses [15,16,17], which may help inform vaccine development. Currently, HST is the dominant transmission and determine the spread of HIV epidemic from high-risk groups to the general population in China. Characterizing the distribution of HIV-1 subtypes through HST will be helpful for prevention and intervention of HIV transmission.

This systematic review and meta-analysis aimed to synthesize the available literature to estimate the prevalence of HIV-1 subtypes via HST in China. A subgroup analysis was performed to further explore the geographical distribution and dynamic change of HIV-1 subtypes over time.

## 2. Methods

### 2.1. Literature Search Strategy

Our literature search was conducted in the following databases: PubMed, Web of Science, China National Knowledge Infrastructure (CNKI), China Biological Medical Database (CBM) and the Chinese Wanfang Database for pertinent articles published from database inception to 25 August 2016. The language were restricted to English and Chinese. Text terms and medical subject headings (MeSH) terms were used in the database search in both English and Chinese. Search terms were listed as follows: (“human immunodeficiency virus” OR “HIV” OR HIV-1” OR “AIDS” OR “acquired immunodeficiency syndrome”) AND (“subtype” OR “genotype” OR “molecular epidemiology”) AND (“China” OR “Chinese mainland”). The references of all included studies were also retrieved to obtain further related studies. Our analyses was undertaken in accordance with the Preferred Reporting Items for Systematic reviews and Meta-Analyses (PRISMA) statement issued in 2009 [18].

### 2.2. Inclusion and Exclusion Criteria

Studies were included if they met the following predetermined criteria: (1) the articles were based on HIV-1 subtypes or molecular epidemiological studies in China; (2) the study population was HIV sero-positive patients, and the route of HIV infection was classified clearly and should contain HST; (3) there were specific laboratory methodologies to classify various HIV-1 subtypes successfully (most analogous studies mainly depend on nucleotide sequences encompassing the amplification of *env*, *gag* and/or *pol* genes by using PCR, and then determine HIV-1 subtype strains by sequencing and phylogenetic tree analysis); (4) they provided the frequency or proportion of different HIV-1 genotypes among heterosexuals; (5) provided reliable information about the study period and location; (6) the study sample size was more than 10 people; (7) full papers were available and published in Chinese or English language.

Studies were excluded for the following reasons: case reports, systematic reviews and meta-analysis, dissertations, and studies that were restricted to only some unique or novel HIV-1 genetic variants were not chosen. Additionally, if the same study data from the same region in some overlapping time periods were published, the article that provided a smaller sample size was excluded. Two investigators separately searched for available publications, included eligible articles and sought consultations from a third source, when necessary.

### 2.3. Data Extraction

On the basis of our established criteria, for each accepted study, the following information were collected: name of the first author, year of publication, study period, geographical locations of the studies, study method to classify the different HIV-1 subtypes, number of successfully identified HIV-1 subtype samples and the frequency or proportion of each HIV-1 subtype through HST. Data was independently extracted from all of the included studies by two investigators, and any discrepancies in data extraction or eligibility assessment were discussed and resolved before reaching a consensus.

### 2.4. Statistical Analysis

Considering the raw proportions of HIV-1 subtypes from extracted data, a Freeman-Tukey double arcsine transformation methodology was implemented to stabilize the variances prior to calculation of the overall estimates [19,20,21]. Metaprop command was executed, and studies with subtype proportions at 0% or 100% were excluded from the meta-analysis. We estimated heterogeneity between studies with Cochran’s *Q* statistic and the *I*^2^ statistic [22,23]. Random effect models were selected for meta-analysis if the significant heterogeneity was tested by *Q* test (*p* < 0.10 was considered to have statistically significant heterogeneity), otherwise fixed-effects models was adopted. We used the *I*^2^ statistic to estimate the degrees of heterogeneity (with values of 25%, 50% and 75% represent respectively low, moderate, and high heterogeneity). Egger’s test was used to assess the publication bias (*p* > 0.05 indicated that no publication bias existed). Subgroup analysis focused on study region, time period, and amplification of HIV-1 gene region. The *χ*^2^ test was used to assess the proportion differences among the subgroups. A sensitive analysis was performed to explore the influence of individual studies on the overall prevalence estimate by serially excluding each study. All analyses were carried out using the meta package of R version 3.3.1 (R Foundation for Statistical Computing, Beijing, China) and SPSS version 20.0 (IBM Inc., New York, NY, USA).

## 3. Results

### 3.1. Study Identification and Selection

**Figure 1:**
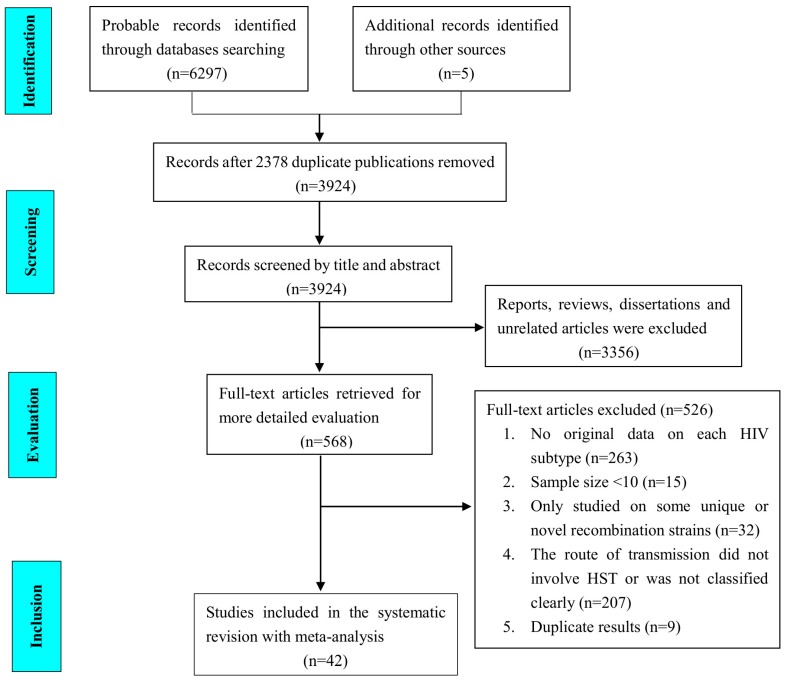
Flowchart of the study identification and selection process.

A flowchart of studies identified by the search is presented in Figure 1. We identified 6297 publications from four electronic databases according to our search strategy and five more publications from literature tracing (PubMed 845; Web of Science 892; CNKI 1856; CBM 1243; Wanfang 1461; literature tracing 5). After removing duplicate publications and initial screening, 568 papers were retrieved in full for a more detailed assessment. Finally, 42 studies met all the proposed inclusion criteria for this meta-analysis.

### 3.2. General Characteristic of the Included Studies

These 42 studies including 17 English articles and 25 Chinese articles published between September 2003 and June 2016, and the study period range was from 1992 to 2014. The study regions covered the following 12 provinces or municipalities: Beijing, Shanghai, Jiangsu, Zhejiang, Tianjin, Hebei, Guangdong, Fujian, Guangxi, Liaoning, Hubei and Yunnan. The number of genotypes identified successfully ranged from 14 to 420, providing a total of 4540 samples. Through analyzing the 42 studies, five major epidemic HIV-1 subtypes were extracted. Additionally, we merged prototypical subtype B and B’ (Thailand variant of subtype B, also referred as Thai B) together due to the lack of detailed categorical data on them among most studies. Details about the included studies are listed in Table 1.

We collated and calculated the raw proportion of different HIV-1 subtypes from all the included studies. The raw proportion of subtype CRF01_AE ranged from 6.45% to 80.08%, CRF07_BC from 1.74% to 59.88%, CRF08_BC from 3.03% to 52.74%, B/B’ from 1.26% to 90.32%, C from 0.34% to 40.70%, and others (including URFs and other subtypes) accounted for 0.50% to 25.00%.

### 3.3. The Overall Proportion of HIV-1 Subtypes

**Figure 2:**
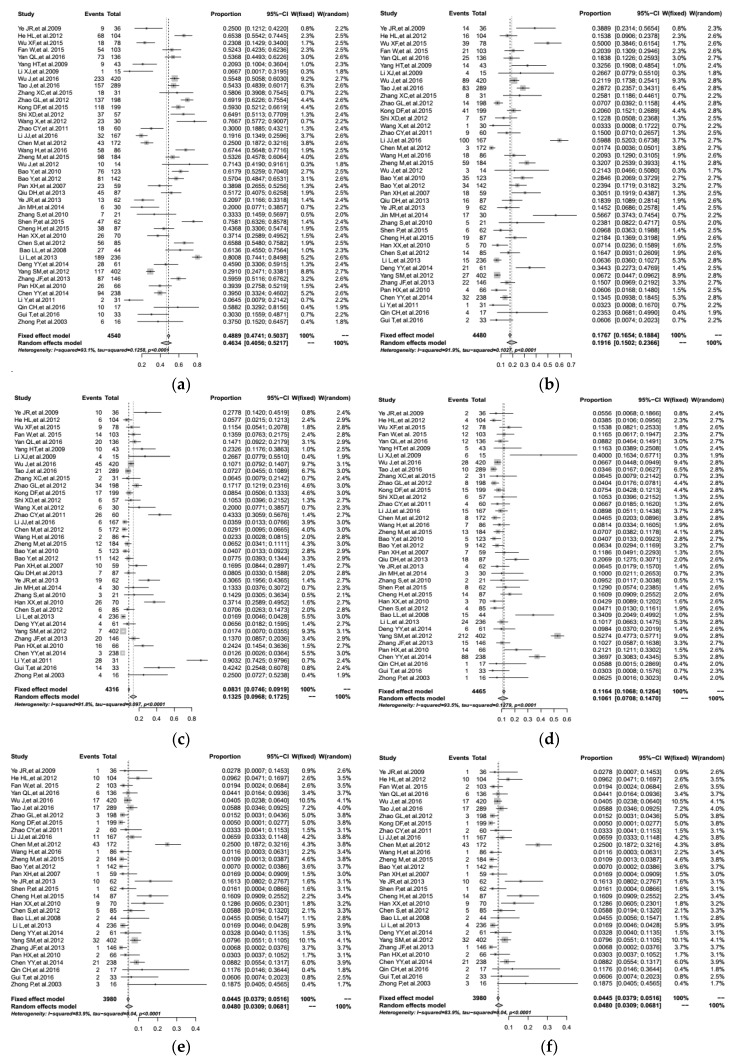
Calculation of the overall proportion by using a meta-analysis method from included studies reporting the proportions of different HIV-1 subtypes via HST in China: (**a**) The overall proportion of CRF01_AE; (**b**) The overall proportion of CRF07_BC; (**c**) The overall proportion of subtype B/B’; (**d**) The overall proportion of CRF08_BC; (**e**) The overall proportion of subtype C; (**f**) The overall proportion of other subtypes.

We used meta-analysis methods with R software to estimate the overall proportion of different HIV-1 subtypes through comprehensive analysis. As shown in Figure 2, the overall proportion of subtype CRF01_AE was 46.34% (95% CI: 40.56–52.17%), CRF07_BC was 19.16% (95% CI: 15.02–23.66%), Subtype B/B’ was 13.25% (95% CI: 9.68–17.25%), CRF08_BC was 10.61% (95% CI: 7.08–14.70%), Subtype C was 4.29% (95% CI: 1.85–7.48%), and the others accounted for 4.80% (95% CI: 3.09–6.81%).

Furthermore, significant heterogeneity was found and the value of I^2^ suggested heterogeneity was substantial for all these subtypes as the following: CRF01_AE (Q = 590.32, *p* < 0.01), CRF07_BC (Q = 481.04, *p* < 0.01), B/B’ (Q = 437.18, *p* < 0.01), CRF08_BC (Q = 586.30, *p* < 0.01), C (Q = 241.87, *p* < 0.01) and others (Q = 180.36, *p* < 0.01). As listed in Supplementary Figures S1 and S2, there was no evidence of obvious publication bias by Egger’s test across studies (*p* > 0.05), excluding the data of subtype B/B’.

### 3.4. Temporal Trend of HIV-1 Subtypes

**Figure 3:**
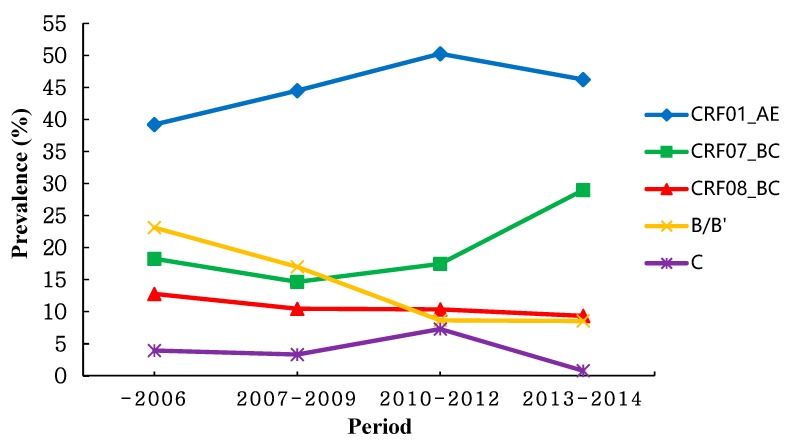
Trend of the prevalence of different HIV-1 subtypes via HST in recent years.

Table 2 shows a subgroup analysis performed by study period (sample collection year) to reflect the dynamic trend of overall prevalence estimates, and for studies whose study period extended for more than 1 year the mid-year was calculated. As shown in Figure 3, the results indicated the trend of CRF08_BC and C was relatively stable. Meanwhile, the overall prevalence of CRF01_AE, CRF07_BC and B/B’ had a striking fluctuation. The prevalence of CRF01_AE and CRF07_BC showed a increasing tendency, while the prevalence of B/B’ decreased over time. Then after 2010, the prevalence of CRF01_AE tended to be a slight decreasing. Additionally, the prevalence of CRF07_BC and CRF08_BC overtook that of B/B’ with the gradual decline in the prevalence of subtype B/B’ since 2010.

### 3.5. Geographic Distribution of HIV-1 Subtypes

**Figure 4:**
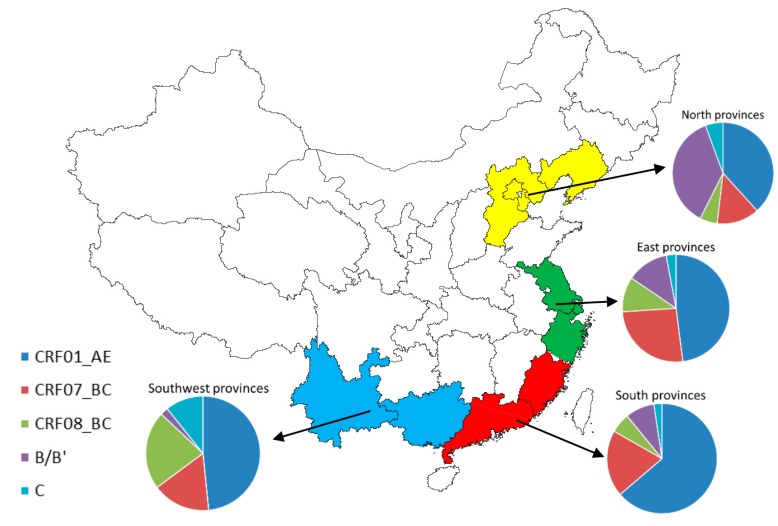
Proportion of HIV-1 subtypes via HST in different geographical provinces.

As shown in Table 2, we classified several provinces based on the adjacent geographical location in China as different subgroups, which included East provinces (Jiangsu, Zhejiang, Shanghai), South provinces (Guangdong, Fujian), North provinces (Beijing, Tianjin, Hebei, Liaoning) and Southwest provinces (Yunnan, Guangxi). The distribution of multiple HIV-1 subtypes in different geographical locations was presented in Figure 4. In East Provinces, CRF01_AE and CRF07_BC were the predominant HIV circulating strains, where they occupied 46.05% (95% CI: 40.44–51.70%) and 24.93% (95% CI: 20.06–30.11%) respectively. In South Provinces, CRF01_AE was predominant strain which has reached 61.75% (95% CI: 57.18–66.21%), and the second was CRF07_BC accounting for 18.97% (95% CI: 12.95–25.80%). In North Provinces, CRF01_AE and subtype B/B’ were the two major epidemic strains. The proportions of corresponded to 35.73% (95% CI: 22.50–50.12%) and 34.48% (95% CI: 29.02–40.14%). In Southwest provinces, CRF01_AE, CRF08_BC and CRF07_BC was the three major circulating strains according to the proportion from high to low commensurately, which accounted for 44.80% (95% CI: 28.17–62.03%), 20.53% (95% CI: 8.51–35.87%) and 15.19% (95% CI: 5.13–29.02%) respectively. There was significant statistical difference among four study regions for the proportion of these epidemic HIV-1 subtypes (*p* < 0.01).

### 3.6. Amplification of HIV-1 Gene Region

With regard to the different amplification and sequencing of HIV-1 gene region (gag/pol/env) from original studies, it was divided into two groups including “only one” and “two or more” regions. Table 1 presents that there was no significant proportion differences on CRF01_AE and CRF08_BC, but the proportion differences on CRF07_BC, B/B’ and C was revealed among two groups (p < 0.01), the overall proportion of CRF07_BC, B/B’ and C was 21.14% (95% CI: 15.58–27.26%), 15.19% (95% CI: 10.15–20.99%), and 2.05% (95% CI: 0.77–3.75%) in “only one” group, 16.23% (95% CI: 10.97–22.24%), 10.75% (95% CI: 6.24–16.19%), and 6.83% (95% CI: 1.42–15.20%) in “two or more” group, respectively

### 3.7. Sensitivity Analysis

**Figure 5:**
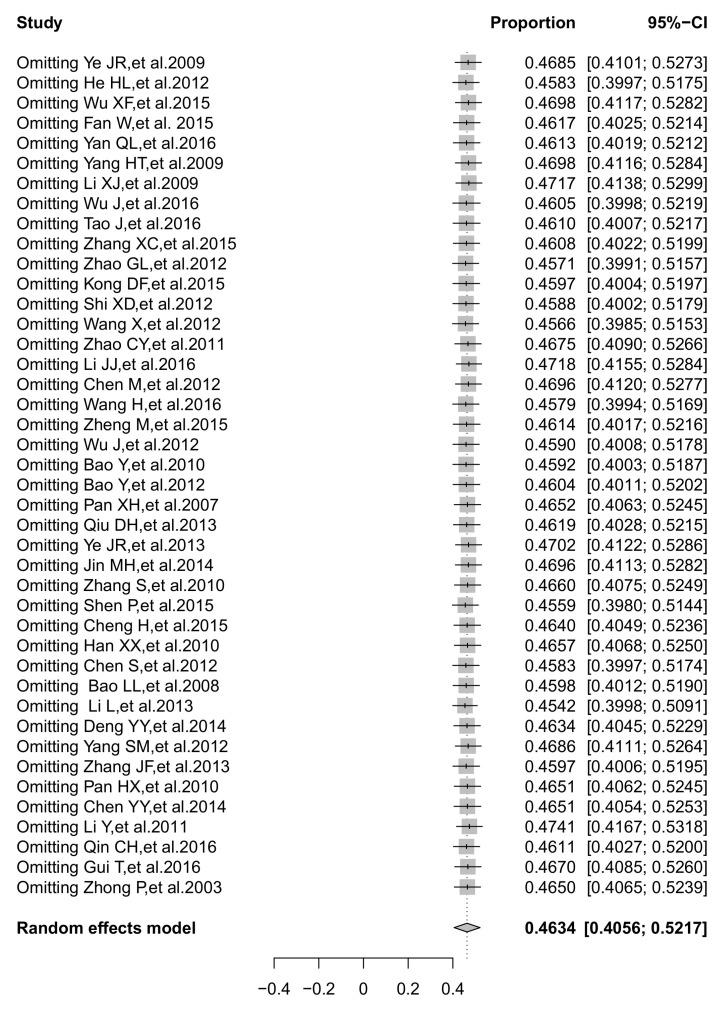
The forest plot of sensitivity analysis of the pooled proportion of CRF01_AE.

The sensitivity analysis results for five HIV-1 strains (CRF01_AE, CRF07_BC, CRF08_BC, B/B’, C) did not significantly alter the overall prevalence after omitting one study at a time. No individual study affected the overall prevalence estimate of CRF01_AE or CRF07_BC or CRF08_BC by more than 1.1%. Particular individual studies were affecting the overall estimate of subtype B/B’ or C >1.5% but <1.8%. One of the sensitivity analysis of CRF01_AE is presented in Figure 5, other results are displayed in Supplementary Figure S3.

## 4. Discussion

Meta-analysis is a statistical method through which data from a number of published studies are pooled to produce relatively reliable data. At present, HST is responsible for the bulk of HIV infection in China. More strikingly, HIV-1 is spreading out of former high-risk groups (MSM, IDUs and FPDs) and introducing into general populations through HST in many regions in China [62,64,66]. Therefore, it is necessary for us to deepen our knowledge of HIV-1 genetic diversity and its distribution among heterosexuals.

We used meta-analysis and subgroup analysis to demonstrate a comprehensive evaluation of the geographic and dynamic change of HIV-1 subtypes through HST over time in China. Additionally, we explored different amplification of HIV gene region whether or not to affect the estimates on prevalence of HIV subtypes, it can provide valuable information for further research. A total of 42 studies were enrolled by meta-analysis method, and the results showed that the major prevalent HIV-1 strains via HST were subtype CRF01_AE, followed by CRF07_BC, B/B’, CRF08_BC and C, this finding was nearly consistent with some previous studies across China based on nationwide molecular epidemiologic studies [14,67,68]. Compared with Zhang’s systematic review [21], the result showed that the circulating HIV-1 subtypes among MSM included CRF01_AE (53.46%), B (28.25%), CRF07_BC (18.66%), and CRF08_BC (5.85%), respectively. Our analysis was similar to that, which possibly due to the existence of high-risk heterosexual behaviors, bisexual transmission networks and some co-circulating conditions among MSM and heterosexuals, especially MSM who was involved in bisexual behaviors [64,69,70]. In the nationwide molecular epidemiologic survey, CRF07_BC (48.5%), CRF08_BC (23.6%) and CRF01_AE (21.2%) were the major HIV-1 strains circulating among IDUs [14], which differed from HIV epidemic in heterosexuals. However, our geographical sub-analysis indicated that the high prevalence of CRF08_BC was restricted to the southwest border of China, such as Yunnan and Guangxi provinces, which are representative for other regions in China where the HIV epidemic has evolved from transmission through needle sharing among IDUs to transmission among immediate sexual contacts. This possibly reflected the existence of high-risk sexual behaviors concurrency between IDUs and heterosexuals. Notably, IDUs and high-risk heterosexual sex populations might facilitate the recombination of HIV strains that are currently present [71]. Similarly, our results showed that the proportion of CRF07_BC was an increasing trend since 2007, this likely attributed to the continual activities between heterosexual behaviors and drug use in these areas where the drug trafficking activities are frequent [14,72,73], especially for the phenomenon of prostitution to support drug use.

In our subgroup analysis, we found that the distribution of HIV-1 subtypes among HST population in different regions in China was not balanced. A relatively higher prevalence of CRF01_AE was found in South provinces, CRF07_BC in East provinces, CRF08_BC and C in Southwest provinces, and B/B’ in North provinces, respectively. On the other hand, the characteristic shown in these areas also reflected that there were certain similarities of circulating HIV-1 subtype distribution among neighboring provinces in China [74], indicating that there might be a cooperative transmission network that could facilitate HIV-1 epidemic within the same population in geographic proximity. In addition, the prevalence of different HIV-1 subtypes has changed constantly in recent years. A strong trend from the results was that the prevalence of CRF01_AE and CRF07_BC increased, whereas the prevalence of subtype B/B’ decreased, and the prevalence of CRF07_BC and CRF08_BC has outstripped that of B/B’ after 2010. These demonstrated that the increasing percentages of CRFs exceeded those of the previously pure subtypes, such as subtype B or C, and CRFs have become the predominant circulating HIV-1 strains, which is consistent with other studies in many Chinese cities [53,75,76]. Of note, although the HIV-1 major prevalent strains in various regions and major high-risk populations were characterized by some differences, these differences tend to be diminished gradually over time, especially in areas where sexual transmission of HIV was predominant. The route of transmission had more pronounced impacts on HIV gene diversity than the geographical locations. This information strongly suggests that unprotected sex between HIV/AIDS infections and its high-risk populations in China are the “incubator” and “accelerator” of the diversity of HIV-1 subtypes in recent years [77]. Therefore, behavioral intervention efforts targeting high-risk populations are urgently needed to curb HIV spreading.

Molecular epidemiological investigation is an important method to analyze and trace the origin and transmission mode of HIV, where multitudinous genotypes are the reflection of evolution history of HIV variation. Our meta-analysis suggested that CRF01_AE, CRF07_BC and CRF08_BC were the three major HIV-1 recombinant strains among heterosexuals. In China, CRF01_AE was detected originally among IDUs who were infected by commercial sex workers from Thailand returning to Yunnan province in 1989 [78]. CRF07_BC and CRF08_BC were first reported from China, the time was presumably traced back to the early 1990s [79]. Of note, a multicenter cohort study suggested CRF01_AE tended to be an independent risk factor for a more rapid HIV-1 progression to AIDS and advanced immunodeficiency, which might be ascribed to higher proportion of X4-tropic viruses in sexually infected patients [15]. Accumulating evidences on a high proportion of X4 tropism and the randomization of HIV transmission among newly diagnosed individuals infected by CRF01_AE strain indicate that CRF01_AE was most likely a severe epidemic in early infections [80,81,82,83,84]. Additionally, researchers used growth kinetic analysis to compare CRF07_BC and subtype B primary isolates. The result indicated that CRF07_BC, as well as infectious recombinant viruses carrying a 7 amino-acid deletion in p6gag had significantly lower replication capacity [85], also suggesting that CRF07_BC might have important impact on disease progression. Currently, a series of SGRs involving CRFs were previously reported among heterosexuals in China, such as CRF01_AE/B [86], CRF01_AE/CRF07_BC [87], etc. Worldwide, CRFs play an increasingly important role in the HIV pandemic [12]. These situations indicate that CRFs contribute critically to the complexity of HIV-1 epidemic. With the economic developments and intensification of population mobility, HIV subtypes will certainly become more complicated which might pose a formidable HIV prevention challenge and highlights the importance of continuous monitoring of HIV-1 CRFs.

For subtype B, a non-recombinant circulating strain, the present study suggested that it had an apparent decrease among heterosexuals in recent years, but geographical sub-analysis marked it as a major epidemic strain in some regions particularly in the North provinces (Liaoning, Hebei) of China. Subtype B and B’ were first identified among IDUs in Yunnan province approximately in 1989. Over time, B’ gradually became the main strain among all subtype B with the subsequent circulating among FDPs and heterosexuals in inland China, replacing the prototypical subtype B [88,89]. However, other types of B strains assumed highly localized distributions. Noticeably, a recent study indicated that subtype B/B’ was associated with higher rate of transmitted drug resistance/drug resistance mutation [16]. Further studies with longer duration are warranted to illustrate this issue. Nevertheless, it could provide important clinical implications for HIV treatment.

Generally, HIV subtypes, transmission clusters and drug resistant mutation can be identified by amplifying and sequencing *pol* gene. However, it is hard to detect the difference between CRF01_AE and CRF15_01B by the sequence of *pol* region, which requires sequencing of the *env* region for differentiation [54,61]. In addition, the newly identified recombinant forms, like CRF51_01B, CRF57_BC or CRF65_cpx, could not be differentiated from subtypes B and C by sequencing only *pol* region [90,91,92]. Recently, the co-circulation of multiple subtypes might provide more chances to generate new inter-subtype recombinants. HIV-1 recombination often occurs in the *env* and *pol* regions, some recombinants may exist yet not easily identified by the analysis of only one gene region. Simultaneous sequencing more than one HIV gene region will be helpful for identification of potentially new recombinant forms. Our study showed that sequencing different HIV gene regions might affect the overall prevalence estimates of CRF07_BC, B/B’ and C, which might be attributed to CRF07_BC originated by insertion of several short segments of subtype B into the backbone of subtype C [63]. Therefore, full or near full-length genome sequencing is required for confirming any novel recombinant forms.

The validity of original studies was affected by many factors, which might influence meta-analysis [93], so interpretation of the results should be done with caution. There are a number of limitations in this study. First, a substantial amount of heterogeneity was observed among the studies. Although strict criteria were applied and potential sources of heterogeneity were explored by subgroup analysis, conclusive results were not fully obtained. The high heterogeneity might be attributed to the confounding effects of the variation in temporal and geographical distribution of the eligible studies, and the small sample size of the included studies may be another reason. Second, the primary studies were based on the analysis of only partial HIV gene fragments including either *gag*, *pol* or *env*. This method offers limited options for identifying the HIV recombinant forms, thus the proportion of recombinant strains may be underestimated. Third, the included studies derived from central and western China data were insufficient, and such data sets could have an impact on the overall results. Finally, some eligible studies were published in Chinese, which have hindered non-Chinese readers from reviewing the original materials.

## 5. Conclusions

In summary, our meta-analysis provides a comprehensive overview on the geographic and temporal trend of HIV-1 subtypes via HST in China. The results showed that CRF01_AE, CRF07_BC, CRF08_BC, and subtype B are currently the predominant circulating subtypes, and the proportion of HIV-1 subtypes has changed dynamically in different regions and over different time periods. Complex and diverse HIV-1 genetic variants underscore that long-term, effective and integrated interventions and preventions are urgently needed. In particular, promotion of consistent condom use is a very important measure to limit HIV transmission.

## Figures and Tables

**Table 1:** General characteristics of the included studies.

First Author, Publication Year [Reference]	Study Period (Mid-Year) *	Location	Gene Amplification Region	Sample Sizes	The Frequency and Proportion of Different HIV-1 Subtypes					
					CRF01_AE	CRF07_BC	CRF08_BC	B/B‘	C	Other Subtypes
Ye, J.R., 2009 [24]	2007	Beijing	*gag*	36	9 (25.00%)	14 (38.89%)	2 (5.56%)	10 (27.78%)	-	1 (2.78%)
He, H.L., 2012 [25]	2009	Guangdong	*env*, *pol*	104	68 (65.38%)	16 (15.38%)	4 (3.85%)	6 (5.77%)	-	10 (9.62%)
Wu, X.F., 2015 [26]	2013	Zhejiang	*gag*	78	18 (23.08%)	39 (50.00%)	12 (15.38%)	9 (11.54%)	-	-
Fan, W., 2015 [27]	2006–2014 (2010)	Jiangsu	*env*, *gag*	103	54 (52.43%)	21 (20.39%)	12 (11.65%)	14 (13.59%)	-	2 (1.94%)
Yan, Q.L., 2016 [28]	2014	Jiangsu	*env*	136	73 (53.68%)	25 (18.38%)	12 (8.82%)	20 (14.71%)	-	6 (4.41%)
Yang, H.T., 2009 [29]	2006	Jiangsu	*env*, *gag*	43	9 (20.93%)	14 (32.56%)	5 (11.63%)	10 (23.26%)	5 (11.63%)	-
Li, X.J., 2009 [30]	1994–2002 (1998)	Yunnan	*pol*, *gag*	15	1 (6.67%)	4 (26.67%)	6 (40.00%)	4 (26.67%)	-	-
Wu, J., 2016 [31]	2007–2013 (2010)	Shanghai	*pol*	420	233 (55.48%)	89 (21.19%)	28 (6.67%)	45 (10.71%)	8 (1.90%)	17 (4.05%)
Tao, J., 2016 [32]	2013	Shanghai	*pol*	289	157 (54.33%)	83 (28.72%)	10 (3.46%)	21 (7.27%)	1 (0.35%)	17 (5.88%)
Zhang, X.C., 2015 [33]	2013	Shanghai	*pol*	31	18 (58.06%)	8 (25.81%)	2 (6.45%)	2 (6.45%)	1 (32.26%)	-
Zhao, G.L., 2012 [34]	1992–2008 (2000)	Guangdong	*env*	198	137 (69.19%)	14 (7.07%)	8 (4.04%)	34 (17.17%)	2 (1.01%)	3 (1.52%)
Kong, D.F., 2105 [35]	2007–2010 (2008–2009)	Guangdong	*env*, *gag*	199	118 (59.30%)	41 (20.60%)	15 (7.54%)	17 (8.54%)	7 (3.52%)	1 (0.50%)
Shi, X.D., 2012 [36]	2010	Guangdong	*env*, *gag*	57	37 (64.91%)	7 (12.28%)	6 (10.53%)	6 (10.53%)	1 (1.75%)	-
Wang, X., 2012 [37]	2010	Tianjin	*gag*	30	23 (76.67%)	1 (3.33%)	-	6 (20.00%)	-	-
Zhao, C.Y., 2011 [38]	2009	Hebei	*env*, *gag*	60	18 (30.00%)	9 (15.00%)	4 (6.67%)	26 (43.33%)	1 (1.67%)	2 (3.33%)
Li, J.J., 2016 [39]	2012–2014 (2013)	Yunnan	*env*	167	32 (19.16%)	100 (59.88%)	15 (8.98%)	6 (35.93%)	3 (1.80%)	11 (6.59%)
Chen, M., 2012 [40]	2011	Yunnan	*env*, *gag*	172	43 (25.00%)	3 (1.74%)	8 (4.65%)	5 (2.91%)	70 (40.70%)	43 (25.00%)
Wang, H., 2016 [41]	2010–2012 (2011)	Guangxi	*pol*	86	58 (67.44%)	18 (20.93%)	7 (8.14%)	2 (2.33%)	-	1 (1.16%)
Zheng, M., 2015 [42]	2013	Shanghai	*pol*	184	98 (53.26%)	59 (32.07%)	13 (7.07%)	12 (6.52%)	-	2 (1.08%)
Wu, J., 2012 [43]	2010	Shanghai	*pol*	14	10 (71.43%)	3 (21.43%)	-	-	1 (7.14%)	-
Bao, Y., 2010 [44]	2009	Guangdong	*env*, *gag*	123	76 (61.79%)	35 (28.46%)	5 (4.07%)	5 (4.07%)	2 (1.63%)	-
Bao, Y., 2012 [45]	2010	Guangdong	*env*, *gag*	142	81 (57.04%)	34 (23.94%)	9 (6.34%)	11 (7.75%)	6 (4.23%)	1 (0.70%)
Pan, X.H., 2007 [46]	2003–2005 (2004)	Zhejiang	*gag*	59	23 (38.98%)	18 (30.51%)	7 (11.86%)	10 (16.95%)	-	1 (1.69%)
Qiu, D.H., 2013 [47]	2011	Zhejiang	*pol*	87	45 (51.72%)	16 (18.39%)	18 (20.69%)	7 (8.05%)	1 (1.15%)	-
Ye, J.R., 2013 [48]	2006–2010 (2008)	Beijing	*gag*	62	13 (20.97%)	9 (14.52%)	4 (6.45%)	19 (30.65%)	7 (11.29%)	10 (16.13%)
Jin, M.H., 2014 [49]	2008–2012 (2010)	Zhejiang	*gag*	30	6 (20.00%)	17 (56.67%)	3 (10.00%)	4 (13.33%)	-	-
Zhang, S., 2010 [50]	2009	Zhejiang	*pol*	21	7 (33.33%)	5 (23.81%)	2 (9.52%)	3 (14.29%)	4 (19.05%)	-
Shen, P., 2015 [51]	2014	Guangxi	*gag*	62	47 (75.81%)	6 (9.68%)	8 (12.90%)	-	-	1 (1.61%)
Cheng, H., 2015 [52]	2012–2013 (2012–2013)	Jiangsu	*env*	87	38 (43.68%)	19 (21.84%)	14 (16.09%)	-	2 (2.30%)	14 (16.10%)
Han, X.X., 2010 [53]	2000–2008 (2004)	Liaoning	*gag*	70	26 (37.14%)	5 (7.14%)	3 (4.29%)	26 (37.14%)	1 (1.43%)	9 (12.86%)
Chen, S., 2012 [54]	2009	Guangdong	*pol*, *env*	85	56 (65.88%)	14 (16.47%)	4 (4.71%)	6 (7.06%)	-	5 (5.88%)
Bao, L.L., 2008 [55]	1996–2005 (2000–2001)	Yunnan	*Pol*, *env*	44	27 (61.36%)	-	15 (34.09%)	-	-	2 (4.54%)
Li, L., 2013 [56]	2009	Guangxi	*gag*, *pol*	236	189 (80.08%)	15 (6.36%)	24 (10.17%)	4 (1.69%)	-	4 (1.69%)
Deng, Y.Y., 2014 [57]	2011–2012 (2011–2012)	Fujian	*env*, *gag*, *pol*	61	28 (45.90%)	21 (34.43%)	6 (9.84%)	4 (6.56%)	-	2 (3.28%)
Yang, S.M., 2012 [58]	2008–2009 (2008–2009)	Yunnan	*env*, *gag*, *pol*	402	117 (29.10%)	27 (6.72%)	212 (52.74%)	7 (1.74%)	7 (1.74%)	32 (7.96%)
Zhang, J.F., 2013 [59]	2009	Zhejiang	*gag*	146	87 (59.59%)	22 (15.07%)	15 (10.27%)	20 (13.70%)	1 (0.68%)	1 (0.68%)
Pan, H.X., 2010 [60]	2008	Zhejiang	*gag*	66	26 (39.39%)	4 (6.06%)	14 (21.21%)	16 (24.24%)	4 (6.06%)	2 (3.03%)
Chen, Y.Y., 2014 [61]	2009–2011 (2010)	Yunnan	*pol*	238	94 (39.50%)	32 (13.44%)	88 (36.97%)	3 (12.61%)	-	21 (8.82%)
Li, Y., 2011 [62]	2007–2008 (2007–2008)	Hubei	*gag*	31	2 (6.45%)	1 (3.23%)	-	28 (90.32%)	-	-
Qin, C.H., 2016 [63]	2011–2013 (2012)	Jiangsu	*env*, *pol*	17	10 (58.82%)	4 (23.53%)	1 (5.88%)	-	-	2 (11.76%)
Gui, T., 2016 [64]	2012	Hebei	*gag*, *pol*	33	10 (30.30%)	2 (6.06%)	1 (3.03%)	14 (42.42%)	4 (12.12%)	2 (6.06%)
Zhong, P., 2003 [65]	1999–2001 (2000)	Shanghai	*env*, *gag*	16	6 (37.50%)	-	1 (6.25%)	4 (25.00%)	2 (12.50%)	3 (18.75%)

Study period (Mid-year) *, if individual studies whose study period extended for more than 1 calendar year, the mid-year was calculated.

**Table 2:** Pooled proportion of different HIV-1 subtypes stratified by study region, amplified gene region.

Subgroups	CRF01_AE				CRF07_BC				CRF08_BC			
	N	Proportion, % (95% CI)	*p ^#^*, *I*^2^	p *	N	Proportion, % (95% CI)	*p ^#^*, *I*^2^	p *	N	Proportion, % (95% CI)	*p ^#^, I*^2^	p *
**Study region**				<0.01				<0.01				<0.01
East provinces	18	46.05 (40.44–51.70)	<0.01, 79.9%		17	24.93 (20.06–30.11)	<0.01, 80.1%		17	10.10 (7.44–13.08)	<0.01, 66.8%	
South provinces	8	61.75 (57.18–66.21)	0.04, 50.5%		8	18.97 (12.95–25.80)	<0.01, 84.2%		8	5.64 (4.21–7.24)	0.39, 4.3%	
North provinces	6	35.73 (22.50–50.12)	<0.01, 83.4%		6	12.67 (5.58–21.85)	<0.01, 75.5%		5	5.22 (2.64–8.48)	0.95, 0.0%	
Southwest provinces	9	44.80 (28.17–62.03)	<0.01, 97.5%		8	15.19 (5.13–29.02)	<0.01, 97.2%		9	20.53 (8.51–35.87)	<0.01, 97.4%	
**Time period**				<0.01				<0.01				0.24
–2006	7	39.18 (22.58–57.09)	<0.01, 91.5%		5	18.25 (7.16–32.65)	<0.01, 88.0%		7	12.77 (4.92–23.17)	<0.01, 84.4%	
2007–2009	13	44.48 (32.00–57.31)	<0.01, 95.9%		13	14.64 (9.98–19.98)	<0.01, 85.3%		12	10.43 (3.03–21.16)	<0.01, 96.8%	
2010–2012	13	50.23 (41.35–59.10)	<0.01, 89.0%		13	17.45 (11.09–24.81)	<0.01, 89.1%		11	10.34 (4.71–17.63)	<0.01, 92.1%	
2013–2014	9	47.55 (36.16–59.07)	<0.01, 93.1%		9	28.96 (19.37–39.75)	<0.01, 92.7%		9	9.32 (6.29–12.82)	<0.01, 68.6%	
**Amplified gene region**				0.08				<0.01				0.55
Only one	24	45.16 (38.14–52.28)	<0.01, 91.2%		24	21.14 (15.58–27.26)	<0.01, 91.8%		21	10.39 (6.94–14.40)	<0.01, 88.2%	
Two or more	18	47.78 (37.70–57.96)	<0.01, 94.5%		16	16.23 (10.97–22.24)	<0.01, 89.5%		18	10.95 (4.63-19.25)	<0.01, 95.7%	
**Subgroups**	**B/B’**				**C**							
	**N**	**Proportion, % (95% CI)**	***p ^#^*** **, *I*^2^**	p *	**N**	**Proportion, % (95% CI)**	***p ^#^*** **, *I*^2^**	p *				
**Study region**				<0.01				<0.01				
East provinces	15	12.09 (9.47–14.96)	<0.01, 57.2%		11	2.83 (0.86–5.59)	<0.01, 71.4%					
South provinces	8	8.28 (5.50–11.52)	<0.01, 62.7%		5	2.28 (1.23–3.58)	0.32, 13.7%					
North provinces	6	34.48 (29.02–40.14)	0.21, 29.0%		4	5.26 (0.87–12.27)	0.02, 69.4%					
Southwest provinces	7	2.07 (0.85–3.69)	0.03, 56.2%		3	9.95 (0.00–38.04)	<0.01, 98.8%					
**Time period**				<0.01				<0.01				
–2006	6	23.10 (15.85–31.18)	0.03, 58.8%		4	3.92 (0.08–11.24)	<0.01, 75.9%					
2007–2009	13	16.98 (8.56–27.39)	<0.01, 95.6%		8	3.29 (1.27–6.02)	<0.01, 70.6%					
2010–2012	11	8.62 (4.58–13.65)	<0.01, 86.1%		7	7.27 (0.28–20.34)	<0.01, 96.3%					
2013–2014	7	8.52 (5.55–12.03)	<0.01, 65.7%		4	0.75 (0.09–1.82)	0.16, 41.1%					
**Amplified gene region**				<0.01				<0.01				
Only one	21	15.19 (10.15–20.99)	<0.01, 92.1%		13	2.05 (0.77–3.75)	<0.01, 65.1%					
Two or more	16	10.75 (6.24–16.19)	<0.01, 90.2%		10	6.83 (1.42–15.20)	<0.01, 94.8%					

Amplified gene region: “Only one” signified that only one HIV-1 gene region (e.g., *gag* or *env* or *pol*) was amplified from include studies. “Two or more” was that simultaneous amplification of two or more HIV-1 genes was analyzed (e.g., *pol* and *env, gag* and *env*, *gag*, *env* and *pol*, etc.). N, number of included studies. *p ^#^*, estimated heterogeneity with Cochran’s *Q* statistic (*p* < 0.10 was deemed to have statistically significant heterogeneity). p *, assessed the proportion differences among the subgroups by *χ*^2^ test (*p* < 0.05 was regarded as statistical significance).

## Supplementary Materials

The following are available online at www.mdpi.com/1660-4601/14/7/830/s1, Figure S1: Funnel plots analysis to detect publication bias, Figure S2: Egger’s test for funnel plots asymmetry, Figure S3: The forest plot of sensitivity analysis of the proportion of different HIV-1 subtypes (CRF07_BC, CRF08_BC, B/B’ and C).

## References

[1] Zeng Y., Fan J., Zhang Q., Wang P.C., Tang D.J., Zhon S.C., Zheng X.W., Liu D.P. (1986). Detection of antibody to LAV/HTLV-III in sera from hemophiliacs in China. AIDS Res..

[2] Xiao Y., Kristensen S., Sun J., Lu L., Vermund S.H. (2007). Expansion of HIV/AIDS in China: Lessons from Yunnan province. Soc. Sci. Med..

[3] Jia Y., Lu F., Sun X., Vermund S.H. (2007). Sources of data for improved surveillance of HIV/AIDS in China. Southeast Asian J. Trop. Med. Public Health.

[4] Wang N., Wang L., Wu Z., Guo W., Sun X., Poundstone K., Wang Y. (2010). Estimating the number of people living with HIV/AIDS in China: 2003-09. Int. J. Epidemiol..

[5] National Center for AIDS/STD Control and Prevention, China CDC (2017). Update on the AIDS/STD epidemic in China and main response in control and prevention in December, 2016. Chin. J. AIDS STD.

[6] Wang L., Ding Z., Qin Q., Cai C., Guo W., Cui Y. (2015). Characteristics of HIV transmission through heterosexual contact in China, 2008–2014. Zhonghua Liuxingbingxue Zazhi.

[7] Roberts J.D., Bebenek K., Kunkel T.A. (1988). The accuracy of reverse transcriptase from HIV-1. Science.

[8] Lemey P., Pybus O.G., Wang B., Saksena N.K., Salemi M., Vandamme A.M. (2003). Tracing the origin and history of the HIV-2 epidemic. Proc. Natl. Acad. Sci. USA.

[9] Sharp P.M., Hahn B.H. (2011). Origins of HIV and the AIDS pandemic. Cold Spring Harb. Perspect. Med..

[10] Vidal N., Peeters M., Mulanga-Kabeya C., Nzilambi N., Robertson D., Ilunga W., Sema H., Tshimanga K., Bongo B., Delaporte E. (2000). Unprecedented degree of human immunodeficiency virus type 1 (HIV-1) group M genetic diversity in the Democratic Republic of Congo suggests that the HIV-1 pandemic originated in Central Africa. J. Virol..

[11] Sharp P.M., Hahn B.H. (2010). The evolution of HIV-1 and the origin of AIDS. Philos. Trans. R. Soc. Lond. Ser. B Biol. Sci..

[12] Hemelaar J., Gouws E., Ghys P.D., Osmanov S. (2011). Global trends in molecular epidemiology of HIV-1 during 2000–2007. AIDS.

[13] Hemelaar J. (2012). The origin and diversity of the HIV-1 pandemic. Trends. Mol. Med..

[14] He X., Xing H., Ruan Y., Hong K., Cheng C., Hu Y., Xin R., Wei J., Feng Y., Hsi J.H. (2012). A comprehensive mapping of HIV-1 genotypes in various risk groups and regions across China based on a nationwide molecular epidemiologic survey. PLoS ONE.

[15] Li Y., Han Y., Xie J., Gu L., Li W., Wang H., Lv W., Song X., Li Y., Routy J.P. (2014). CRF01_AE subtype is associated with X4 tropism and fast HIV progression in Chinese patients infected through sexual transmission. AIDS.

[16] Li Y., Gu L., Han Y., Xie J., Wang H., Lv W., Song X., Li Y., Iwamoto A., Ishida T. (2015). HIV-1 subtype B/B’ and baseline drug resistance mutation are associated with virologic failure: A multicenter cohort study in China. J. Acquir. Immune Defic. Syndr..

[17] Sui H., Gui T., Jia L., Guo W., Han J., Liu Y., Bao Z., Li H., Li J., Li L. (2014). Different frequencies of drug resistance mutations among HIV-1 subtypes circulating in China: A comprehensive study. PLoS ONE.

[18] Liberati A., Altman D.G., Tetzlaff J., Mulrow C., Gotzsche P.C., Ioannidis J.P., Clarke M., Devereaux P.J., Kleijnen J., Moher D. (2009). The PRISMA statement for reporting systematic reviews and meta-analyses of studies that evaluate health care interventions: Explanation and elaboration. J. Clin. Epidemiol..

[19] Miller J.J. (1978). The inverse of the Freeman-Tukey double arcsine transformation. Am. Stat..

[20] Cassone A. (2012). Prevalence of tuberculosis, hepatitis C virus, and HIV in homeless people: A systematic review and meta-analysis. Pathog. Glob. Health.

[21] Zhang L., Wang Y.J., Wang B.X., Yan J.W., Wan Y.N., Wang J. (2015). Prevalence of HIV-1 subtypes among men who have sex with men in China: A systematic review. Int. J. STD AIDS.

[22] Higgins J.P., Thompson S.G., Deeks J.J., Altman D.G. (2003). Measuring inconsistency in meta-analyses. BMJ.

[23] Higgins J.P., Thompson S.G. (2002). Quantifying heterogeneity in a meta-analysis. Stat. Med..

[24] Ye J.R., Li Y., Bai L.S. (2009). Sequence and subtype analysis of gag gene of isolates confirmed by HIV-1 infections in Beijing in 2007. Virology.

[25] He H.L., Xu F.l., Cai W.P., Chen X.J., He J.Y., Jia W.D. (2012). Molecular epidemiology of HIV-1 in Guangdong province. Guangdong Med..

[26] Wu X.F., Cha Y.F., Ji L., Jing M.H. (2015). Gene subtype analysis of HIV-1 strains isolated from different population groups in Huzhou. Chin. J. Health Lab. Technol..

[27] Fan W., Yan L.Q., Huan X.P. (2015). Study of spreading mode and molecular epidemiological characteristcs of heterosexual transmitted HIV in Huaian. Jiangsu J. Prev. Med..

[28] Yan L.Q., Yang P.F., Guo H.X. (2016). Epidemiological features and molecualr evolution of HIV-1 through heterosexual contact in Huaian. Pathog. Biol..

[29] Yang H.T., Xu X.Q., Qiu T., Huan X.P., Hu H.Y., Guo H.X. (2009). Molecular epidemiology of the human immunodeficiency virus-1 isolated from patients confirmed lately in Jiangsu province. ACTA Univ. Med. Nanjing.

[30] Li X.J., Xia X.S., Cao C.M. (2009). Molecular epidemiology of HIV-1 transmission among heterosexuals in Kunming. Chin. J. Derm. Vener..

[31] Wu J., Wang X.Q., Zhou Y.Q., Yu X.L., Gai J., Yuan J.L., Zheng M., Tao J. (2016). Molecular epidemiological characteristics of HIV-1 in Shanghai, 2007–2013. Chin. J. AIDS STD.

[32] Tao J., Yu X.L., Zhang J. (2016). Molecular epidemiological survey on HIV-1 subtypes in people with heterosexual transmission in Shanghai. J. Diagn. Concepts Pract..

[33] Zhang X.C., Lin Q., Wang X.G., Fan Y. (2015). Molecular epidemiological study of HIV-1 in Minghang District of Shanghai. Chin. Prev. Med..

[34] Zhao G.L., Yu W., Zhang J.J., Chen L., Feng T.J., Wang F. (2012). Study on the molecular-epidemiological characteristics of HIV-1 in Shenzhen, 1992–2008. Zhonghua Liuxingbingxue Zazhi.

[35] Kong D.F., Wang X.H., Qin Y.M. (2015). Molecular epidemiological characteristics of 528 HIV-1 infected cases in Shenzhen. Chin. Trop. Med..

[36] Shi X.D., Chen L., Yang Z.R., Wang X.H., Zhao J., Wang F. (2012). Molecular epidemiology of HIV-1 subtypes in Shenzhen of 2010. Chin. Trop. Med..

[37] Wang X., Zheng M.N., Guo Y. (2012). The genetic subtype of HIV-1 in different groups of HIV-1 infected person in Tianjin. Chin. J. Dis. Control Prev..

[38] Zhao C.Y., Li Q.M., Zhao H.R., Lu X.L. (2011). Study on subtypes distribution and characteristics for HIV-1 infection among MSM and heterosexuals. Chin. J. Dis. Control Prev..

[39] Li J.J., Li H.Q., Li J.Y., Yang S.M. (2016). Characteristics and distribution of HIV-1 subtypes in Yunnan province. Chin. J. AIDS STD.

[40] Chen M., Yao S.T., Ma Y.L., He X., Wang J.B. (2012). Distribution of HIV-1 subtypes among different populations in Dehong prefecture, Yunnan province in 2011. Zhonghua Liuxingbingxue Zazhi.

[41] Wang H., Liang B.Y., Zhou B., Jiang J.J. (2016). Distribution of subtypes of pol gene in HIV-1 epidemic strains in Guangxi Zhuang Autonomous Region, 2010–2012. Chin. J. Prev. Med..

[42] Zheng M., Wu J., Ni Y.Q., Zhou Y.Q. (2015). Molecular epidemiology of HIV-1 in Shanghai in 2013. Chin. J. Infect. Dis..

[43] Wu J., Xue Y.L., Kang L.Y. (2012). Molecular epidemiology and drug resistance: Survey on HIV-1 in Luwan District of Shanghai. J. Diag. Concepts Pract..

[44] Bao Y., Wang X.H., Chen L., He T.P. (2010). Analysis on HIV-1 in homosexual and heterosexual transmission in molecular epidemiology in Shenzhen. Chin. J. Prev. Med..

[45] Bao Y., Wang X.H. (2012). The relationship between distribution of different subtypes of HIV-1 and the transmission routes in Shenzhen. Chin. J. Infect. Dis..

[46] Pan X.H., Yao Y.P., Xia S.C., Yang J.Z., Guo Z.H. (2007). Molecular epidemiology of HIV infection cases in 2003–2005 in Zhejiang province. Chin. J. AIDS STD.

[47] Qiu D.H., Wen J., Shen W.W., Chen X.K. (2013). Molecular epidemiology among newly-diagonsed HIV-1 infections in Taizhou, Zhejiang province, 2011. Dis. Surveil..

[48] Ye J.R., Su X.L., Yu S.Q. (2013). Molecular epidemiological characteristics of HIV-1 strains isolated from newly diagnosed MSM subjects (2006–2010) in Beijing, China. Zhonghua Liuxingbingxue Zazhi.

[49] Jin M.H., Wu X.F., Yang Z.R. (2014). Analysis of subtypes of HIV-1 gag gene through sexually transmission in Huzhou. Chin. J. AIDS STD.

[50] Zhang S., Zhang J.F., Gao H., Jiao S.L. (2010). Molecualr epidemiology on HIV in Ningbo in 2009. Chin. J. HLT.

[51] Shen P., Su Q.J., Huang Y.Y., Jiang L.P., Zhang D.Y., Guo J.C., Wei J.L., Xu H.Z. (2015). Modecular epidemiological study of HIV-1 in Qinann district of Qinzhou. Chin. J. RHSA.

[52] Cheng H., Yuan R., Xie Y., Ji Y.Y., Xiu F.F., Wang B. (2015). A molecular epidemiological study on the human immunodeficiency virus infection in Wuxi city. Chin. J. Dis. Control Prev..

[53] Han X., Dai D., Zhao B., Liu J., Ding H., Zhang M., Hu Q., Lu C., Goldin M., Takebe Y. (2010). Genetic and epidemiologic characterization of HIV-1 infection in Liaoning province, China. J. Acquir. Immune Defic. Synd..

[54] Chen S., Cai W., He J., Vidal N., Lai C., Guo W., He H., Chen X., Fu L., Peeters M. (2012). Molecular epidemiology of human immunodeficiency virus type 1 in Guangdong province of Southern China. PLoS ONE.

[55] Bao L., Vidal N., Fang H., Deng W., Chen S., Guo W., Qin C., Peeters M., Delaporte E., Andrieu J.M. (2008). Molecular tracing of sexual HIV type 1 transmission in the Southwest border of China. AIDS Res. Hum. Retrovir..

[56] Li L., Chen L., Liang S., Liu W., Li T., Liu Y., Li H., Bao Z., Wang X., Li J. (2013). Subtype CRF01_AE dominate the sexually transmitted human immunodeficiency virus type 1 epidemic in Guangxi, China. J. Med. Virol..

[57] Deng Y., Zhang C., Yan Y., Yan P., Wu S. (2014). Genetic subtype and epidemiological feature of HIV-1 circulating strains among recently infected patients in Fujian province. Zhonghua Liuxingbingxue Zazhi.

[58] Yang S.M., Li H.Q., Chen L.L., Li L., Liu Y.J., Zhong M., Li J.J., Yang B.H., Gao L., Fan Y.S. (2012). HIV-1 subtype and the distribution in Yunnan province. Zhonghua Liuxingbingxue Zazhi.

[59] Zhang J.F., Pan X.H., Ding X.B., Chen L., Guo Z.H., Xu Y., Huang J.J. (2013). Molecular epidemiological study on HIV/AIDS under the follow-up program in Zhejiang province in 2009. Zhonghua Liuxingbingxue Zazhi.

[60] Pan X.H., Zhang J.F., Yao Y.P., Xu Y., Yang J.Z., Guo Z.H. (2010). Subtype and transmission of HIV strain in both HIV infected spouses in Zhejiang province. Zhonghua Liuxingbingxue Zazhi.

[61] Chen Y., Chen S., Kang J., Fang H., Dao H., Guo W., Lai C., Lai M., Fan J., Fu L. (2014). Evolving molecular epidemiological profile of human immunodeficiency virus 1 in the Southwest border of China. PLoS ONE.

[62] Li Y., Takebe Y., Yang J., Zhang W., Yang R. (2011). High prevalence of HIV type 1 subtype B’ among heterosexuals in Western Hubei, Central China: Bridging the epidemic into the general population. AIDS Res. Hum. Retrovir..

[63] Qin C., Zhang P., Zhu W., Hao F., Gu A., Fen P., Zhu X., Du H. (2016). HIV-1 diversity in infected individuals in Suzhou and Suqian, China. SpringerPlus.

[64] Gui T., Lu X., Li H., Li T., Liu Y., Bao Z., Li L., Li J. (2016). HIV-1 is spreading out of former high-risk population through heterosexual transmission in Hebei, China. Curr. HIV Res..

[65] Zhong P., Kang L., Pan Q., Konings F., Burda S., Ma L., Xue Y., Zheng X., Jin Z., Nyambi P. (2003). Identification and distribution of HIV type 1 genetic diversity and protease inhibitor resistance-associated mutations in Shanghai, P. R. China. J. Acquir. Immune Defic. Syndr..

[66] Yao X., Wang H., Yan P., Lu Y., Lin H., Chen L., Ng J., Lau E., Liu L., Wu J. (2012). Rising epidemic of HIV-1 infections among general populations in Fujian, China. J. Acquir. Immune Defic. Syndr..

[67] Su Y., Liu H., Wu J., Zhu L., Wang N. (2014). Distribution of HIV-1 genotypes in China: A systematic review. Zhonghua Liuxingbingxue Zazhi.

[68] Li X., Li W., Zhong P., Fang K., Zhu K., Musa T.H., Song Y., Du G., Gao R., Guo Y. (2016). Nationwide trends in molecular epidemiology of HIV-1 in China. AIDS Res. Hum. Retrovir..

[69] Lin H., Ding Y., Liu X., Wu Q., Shen W., He N. (2015). High prevalence of HIV infection and bisexual networks among a sample of men who have sex with men in Eastern China. PLoS ONE.

[70] Yun K., Xu J.J., Reilly K.H., Zhang J., Jiang Y.J., Wang N., Shang H. (2011). Prevalence of bisexual behaviour among bridge population of men who have sex with men in China: A meta-analysis of observational studies. Sex. Transm. Infect..

[71] Wei H., Xing H., Hsi J.H., Jia M., Feng Y., Duan S., He C., Yao S., Ruan Y., He X. (2015). The sexually driven epidemic in youths in China’s Southwestern border region was caused by dynamic emerging multiple recombinant HIV-1 strains. Sci. Rep..

[72] Lim R.B., Wong M.L., Tan P.H., Govender M. (2015). Heterosexual men who patronise entertainment establishments versus brothels in an asian urban setting—which group practises riskier sexual behaviours?. BMC Public Health.

[73] Hittner J.B. (2016). Meta-analysis of the association between methamphetamine use and high-risk sexual behavior among heterosexuals. Psychol. Addict. Behav. J. Soc. Psychol. Addict. Behav..

[74] Tao Y.L., Zhao X.T., Tang Y.F., He N. (2013). A systematic review of temporal and geographical distributions of HIV genotypes in China during 2000–2012. Chin. J. Dis. Control Prev..

[75] Li L., Lu X., Li H., Chen L., Wang Z., Liu Y., Bao Z., Li T., Tian C., Liu H. (2011). High genetic diversity of HIV-1 was found in men who have sex with men in Shijiazhuang, China. Infect. Genet. Evol. J. Mol. Epidemiol. Evol. Genet. Infect. Dis..

[76] Zhang Y., Lu L., Ba L., Liu L., Yang L., Jia M., Wang H., Fang Q., Shi Y., Yan W. (2006). Dominance of HIV-1 subtype CRF01_AE in sexually acquired cases leads to a new epidemic in Yunnan province of China. PLoS Med..

[77] Wang N., Zhong P. (2015). Molecular epidemiology of HIV in China: 1985–2015. Zhonghua Liuxingbingxue Zazhi.

[78] Yu X.F., Chen J., Shao Y., Beyrer C., Lai S. (1998). Two subtypes of HIV-1 among injection-drug users in Southern China. Lancet.

[79] Feng Y., Takebe Y., Wei H., He X., Hsi J.H., Li Z., Xing H., Ruan Y., Yang Y., Li F. (2016). Geographic origin and evolutionary history of China's two predominant HIV-1 circulating recombinant forms, CRF07_BC and CRF08_BC. Sci. Rep..

[80] Parczewski M., Leszczyszyn-Pynka M., Witak-Jedra M., Maciejewska K., Myslinska S., Urbanska A. (2015). The temporal increase in HIV-1 non-R5 tropism frequency among newly diagnosed patients from northern poland is associated with clustered transmissions. J. Int. AIDS Soc..

[81] To S.W., Chen J.H., Wong K.H., Chan K.C., Chen Z., Yam W.C. (2013). Determination of the high prevalence of dual/mixed- or X4-tropism among HIV type 1 CRF01_AE in Hong Kong by genotyping and phenotyping methods. AIDS Res. Hum. Retrovir..

[82] Hedskog C., Mild M., Albert J. (2012). Transmission of the X4 phenotype of HIV-1: Is there evidence against the “random transmission” hypothesis?. J. Infect. Dis..

[83] Li X., Xue Y., Cheng H., Lin Y., Zhou L., Ning Z., Wang X., Yu X., Zhang W., Shen F. (2015). HIV-1 genetic diversity and its impact on baseline CD4+T cells and viral loads among recently infected men who have sex with men in Shanghai, China. PLoS ONE.

[84] Li X., Xue Y., Zhou L., Lin Y., Yu X., Wang X., Zhen X., Zhang W., Ning Z., Yue Q. (2014). Evidence that HIV-1 CRF01_AE is associated with low CD4+T cell count and CXCR4 co-receptor usage in recently infected young men who have sex with men (MSM) in Shanghai, China. PLoS ONE.

[85] Huang S.W., Wang S.F., Lin Y.T., Yen C.H., Lee C.H., Wong W.W., Tsai H.C., Yang C.J., Hu B.S., Lin Y.H. (2014). Patients infected with CRF07_BC have significantly lower viral loads than patients with HIV-1 subtype B: Mechanism and impact on disease progression. PLoS ONE.

[86] Guo H., Hu H., Zhou Y., Huan X., Qiu T., Fu G., Lu J., Wang X. (2014). The identification of a novel HIV-1 CRF01_AE/B recombinant using the near full length genome in Jiangsu province, China. AIDS Res. Hum. Retrovir..

[87] Li T., Sun G., Jia D., Sun C., Wang Z., Liu S., Liu Y., Li H., Wang X., Li J. (2016). Near full-length genome sequences of two novel HIV-1 recombinant forms detected in Henan province, China. AIDS Res. Hum. Retrovir..

[88] Graf M., Shao Y., Zhao Q., Seidl T., Kostler J., Wolf H., Wagner R. (1998). Cloning and characterization of a virtually full-length HIV type 1 genome from a subtype B’-Thai strain representing the most prevalent B-clade isolate in China. AIDS Res. Hum. Retrovir..

[89] Li Z., He X., Wang Z., Xing H., Li F., Yang Y., Wang Q., Takebe Y., Shao Y. (2012). Tracing the origin and history of HIV-1 subtype B' epidemic by near full-length genome analyses. AIDS.

[90] Ng O.T., Eyzaguirre L.M., Carr J.K., Chew K.K., Lin L., Chua A., Leo Y.S., Redd A.D., Quinn T.C., Laeyendecker O. (2012). Identification of new CRF51_01B in singapore using full genome analysis of three HIV type 1 isolates. AIDS Res. Hum. Retrovir..

[91] Wei H., Liu Y., Feng Y., Hsi J., Xing H., He X., Liao L., Yutaka T., Li J., Shao Y. (2014). Genome sequence of a novel HIV-1 circulating recombinant form (CRF57_BC) identified from Yunnan, China. AIDS Res. Hum. Retrovir..

[92] Feng Y., Wei H., Hsi J., Xing H., He X., Liao L., Ma Y., Ning C., Wang N., Takebe Y. (2014). Identification of a novel HIV type 1 circulating recombinant form (CRF65_cpx) composed of CRF01_AE and subtypes B and C in Western Yunnan, China. AIDS Res. Hum. Retrovir..

[93] Zwahlen M., Renehan A., Egger M. (2008). Meta-analysis in medical research: Potentials and limitations. Urol. Oncol..

