# Supplementary figures and images for: Geographic Distribution and Temporal Trends of HIV-1 Subtypes through Heterosexual Transmission in China: A Systematic Review and Meta-Analysis
Source: Int J Environ Res Public Health. 2017 Jul 24;14(7):830. doi: 10.3390/ijerph14070830 (PMC5551268; doi:10.3390/ijerph14070830)

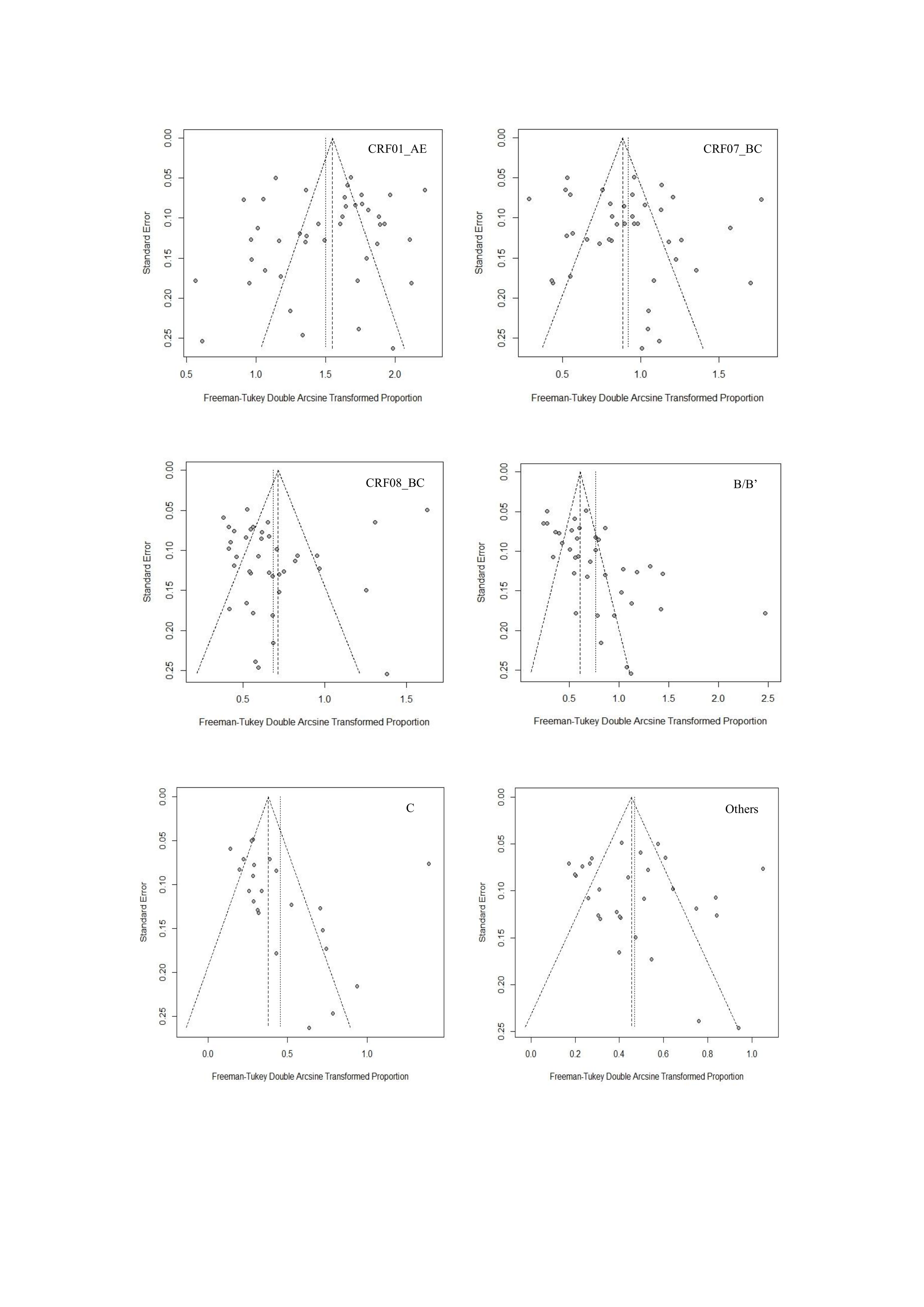

Supplement: Supplementary file 1 [file ijerph-14-00830-s001.zip › Supplementary Files/Figure S1.tif]

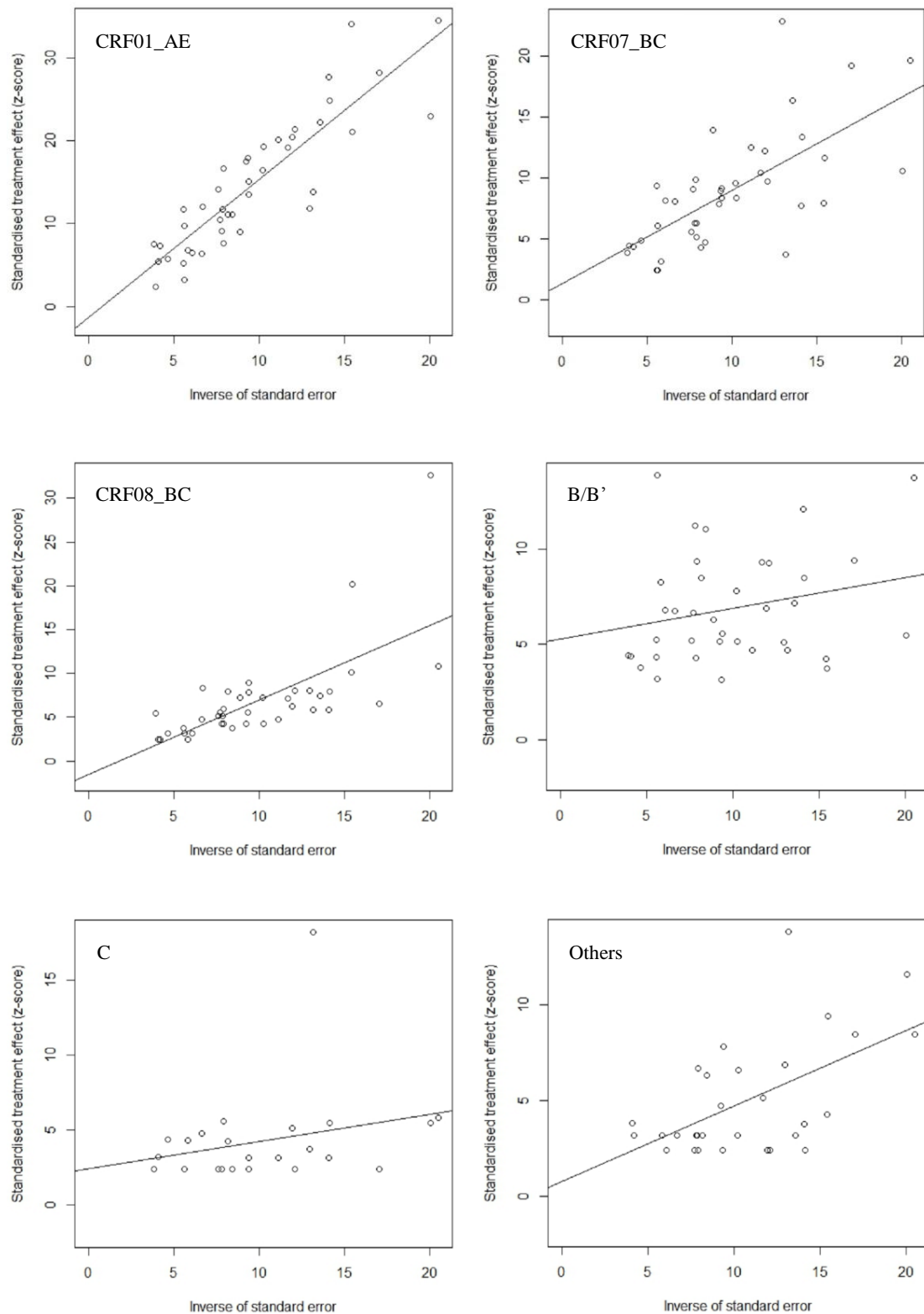

**S2 Fig. Egger's linear regression test of funnel plots asymmetry**

Supplement: Supplementary file 1 [file ijerph-14-00830-s001.zip › Supplementary Files/Figure S2.pdf]

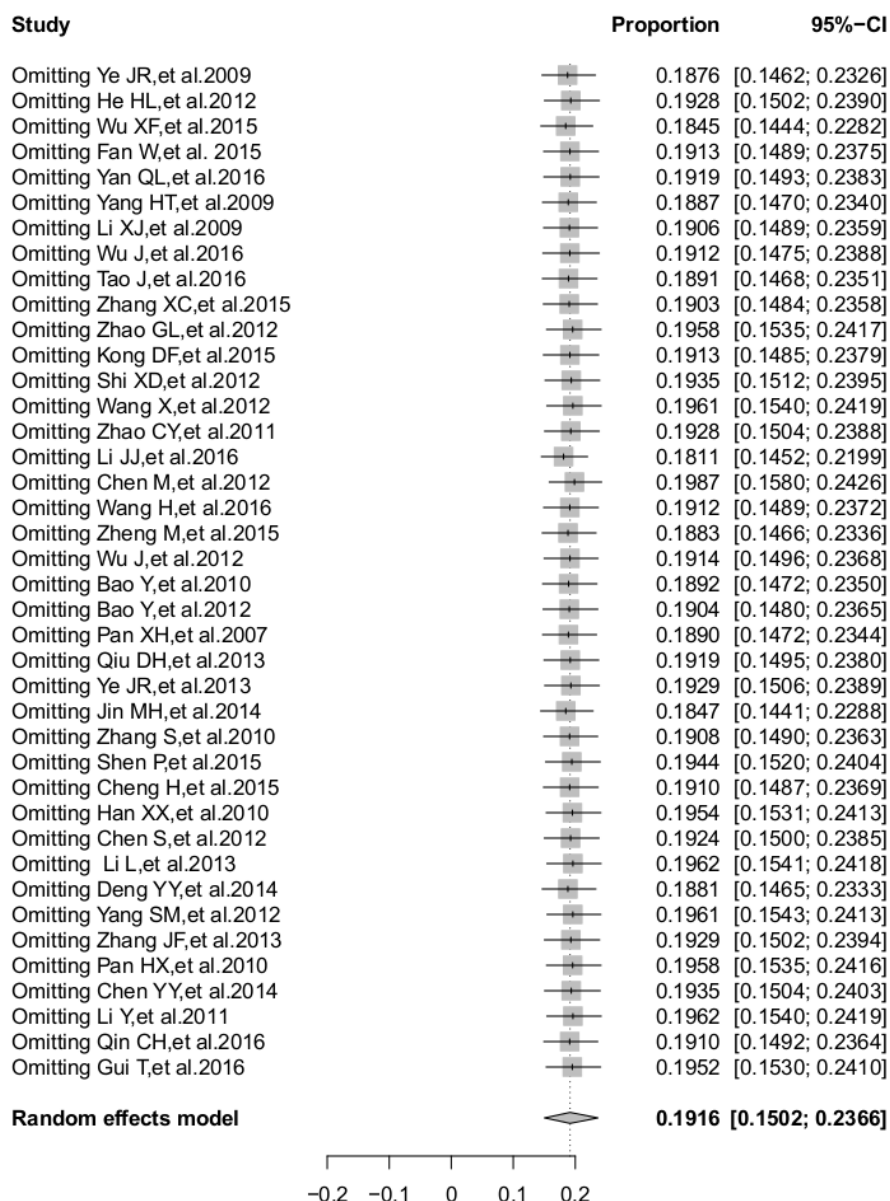

CRF07\_BC

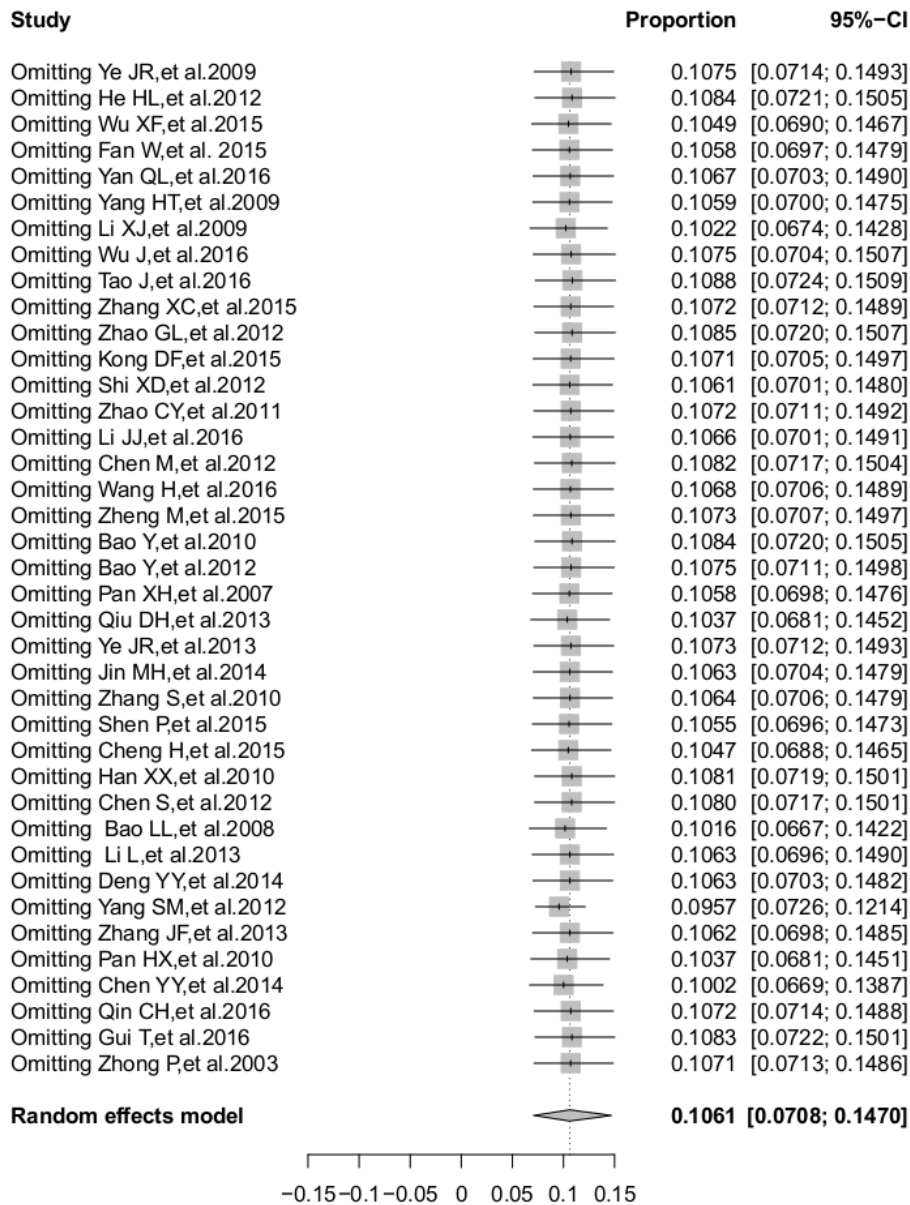

**CRF08\_BC**

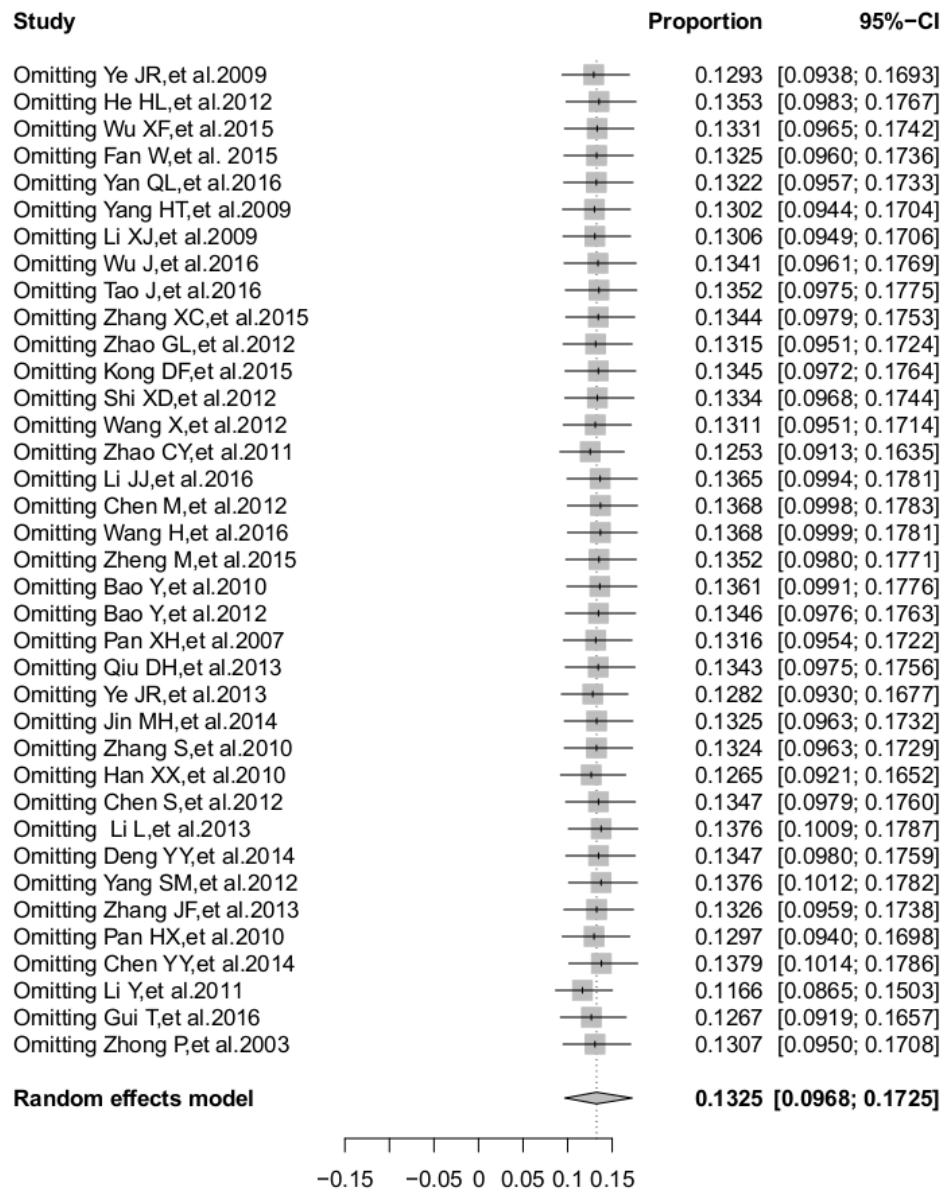

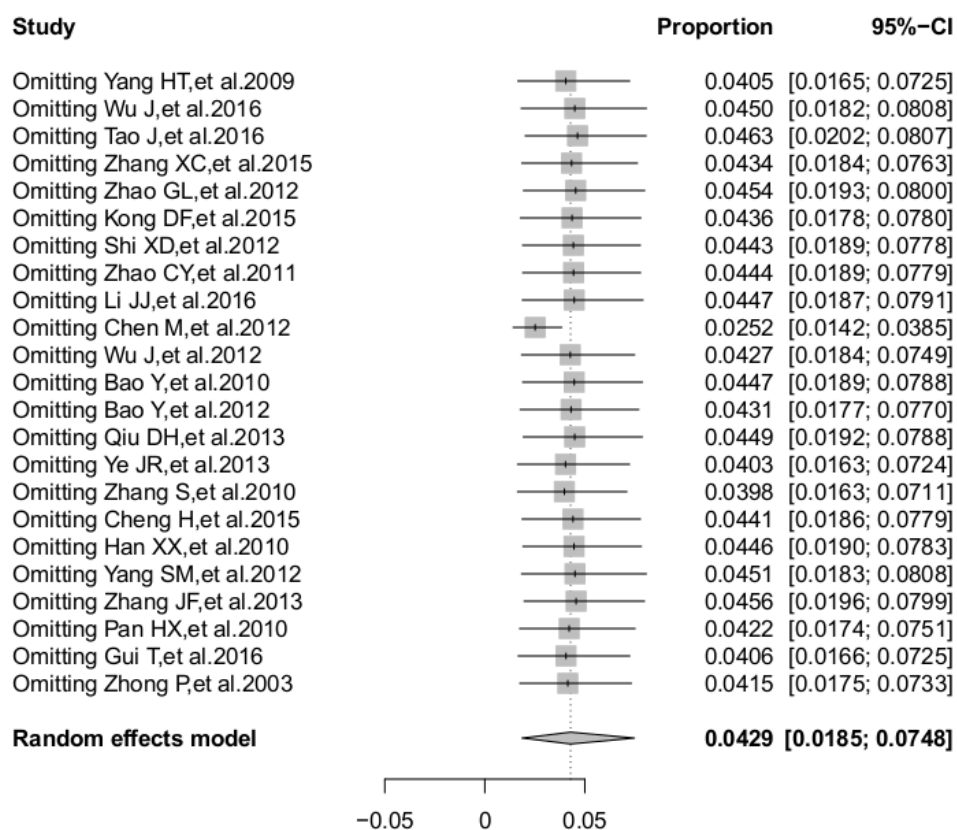

## Subtype C

Supplement: Supplementary file 1 [file ijerph-14-00830-s001.zip › Supplementary Files/Figure S3.pdf]
